# Proximity under Threat: The Role of Physical Distance in Intergroup Relations

**DOI:** 10.1371/journal.pone.0159792

**Published:** 2016-07-28

**Authors:** Y. Jenny Xiao, Michael J. A. Wohl, Jay J. Van Bavel

**Affiliations:** 1 Department of Psychology, New York University, New York City, New York, United States of America; 2 Department of Psychology, Carleton University, Ottawa, Ontario, Canada; Örebro University, SWEDEN

## Abstract

Throughout human history, social groups have invested immense amounts of wealth and time to keep threatening out-groups at a distance. In the current research, we explored the relationship between intergroup threat, physical distance, and discrimination. Specifically, we examined how intergroup threat alters estimates of physical distance to out-groups and how physical proximity affects intergroup relations. Previous research has found that people judge threatening out-groups as physically close. In Studies 1 and 2, we examined ways to attenuate this bias. In Study 1 a secure (vs. permeable) US-Mexico border reduced the estimated proximity to Mexico City among Americans who felt threatened by Mexican immigration. In Study 2, intergroup apologies reduced estimates of physical proximity to a threatening cross-town rival university, but only among participants with cross-group friendships. In Study 3, New York Yankees fans who received an experimental induction of physical proximity to a threatening out-group (Boston Red Sox) had a stronger relationship between their collective identification with the New York Yankees and support for discriminatory policies toward members of the out-group (Red Sox fans) as well as how far they chose to sit from out-group members (Red Sox fans). Together, these studies suggest that intergroup threat alters judgment of physical properties, which has important implications for intergroup relations.

## Introduction

The Great Wall of China—stretching over 5,000 miles—is one of the greatest feats of human engineering. The wall was constructed for the sole purpose of protection from invading Mongolian nomads. The Great Wall not only cost a great fortune, but also agricultural lands and even human lives. From the Great Wall of China to the Border Fence between Mexico and the United States, humans have invested immense amounts of blood and treasure to keep threatening out-groups at a distance. Despite the enormous costs social groups are clearly willing to incur to erect walls, little is known about the psychological function these intergroup barriers serve. Here, we examine how intergroup threat may fundamentally alter people’s judgment of the social and physical landscape (e.g., distance) that exists between the in-group and out-group and the implications for intergroup discrimination. Although research has provided considerable insight into the antecedents and consequences of intergroup threat [1–6], little research has examined the role perceptual representations and judgments of physical reality play in the domain of intergroup relations [7–9]. In this paper, we examined the impact of physical proximity on intergroup relations and how to ameliorate these biased representations and judgments.

The notion that distance judgments impact intergroup relations builds on the “New Look” in perception, which argues values and needs organize people’s visual representations of the physical world [10–12]. According to this approach, the threat people experience should provide motivational influences on their perceptual representation and judgment. Threatening objects tend to be judged closer by subjective estimation than by an objective measurement, in part because it is functionally adaptive to be attuned to immanent threats in the environment [13, 14]. In a similar way, people may judge threatening out-groups as subjectively closer to the in-group than an objectively equidistant non-threatening out-group to help prepare them for impending intergroup conflict [7]. Here we assessed whether elevating in-group security—by building secure barriers between groups or receiving offers of apology from the transgressing out-group—can circumvent the psychological processes that hinder positive intergroup relations. We also manipulated the physical proximity of a threatening out-group to assess the downstream consequences, such as support for discriminatory policies against out-group members.

### Proximity Under Threat

From a biological standpoint, responding to potential threats as if they were clear and present dangers is typically adaptive [15]. Imagine, for example, a hiker who encounters a curved object behind a log on the hiking path. It is far better—for the purpose of survival—to treat a benign twig as a snake and generate a defensive reaction than to ignore a potentially dangerous snake and continue walking blithely along the path [16]. In a similar manner, people tend to represent perceived threatening objects as more physically immediate. For example, people who have a spider phobia are more likely to perceive spiders as physically larger and moving more quickly (“looming”) towards them compared to those who are less fearful of spiders [17, 18]. Moreover, anxiety-prone people perceive negative emotional stimuli as if seen from a closer perspective [19]. Perceived threats reduce body motion, decrease heart rate and increase anxiety in humans—the same physiological reactions found in animals (i.e., freezing) when threatened by predators [20]. These sorts of responses are typically adaptive because they trigger a cascade of reactions that prepare the body for appropriate action [21, 22]. Here, we examine these processes in the domain of intergroup relations.

Although social psychological research has extensively examined the antecedents and consequences of intergroup threat [4, 5, 23], this literature has been relatively silent on the role physical distance plays in intergroup relations. However, research on the perceptual consequences of biological threat may provide a useful framework for understanding reactions to social threats [20]. For instance, a recent paper found that threatening people were judged as physically closer than people who signaled disgust or displayed no affective signal [13]. Presumably, perceiving a threatening person as closer may help people navigate their social environment by preparing a fight or flight response [24]. Just as threatening objects tend to be judged closer by subjective estimation than by an objective measurement [13, 14], locations of a threatening out-group are estimated as subjectively closer to the in-group than a non-threatening out-group [7]. Moreover, the presence (versus absence) of fellow in-group members reduces the effects of intergroup threat on perceived proximity—likely because the presence of in-group members provides a sense of security, which reduces intergroup threat [25]. Taken together, it appears that intergroup threat influences how people judge the physical world.

**Fig 1:**
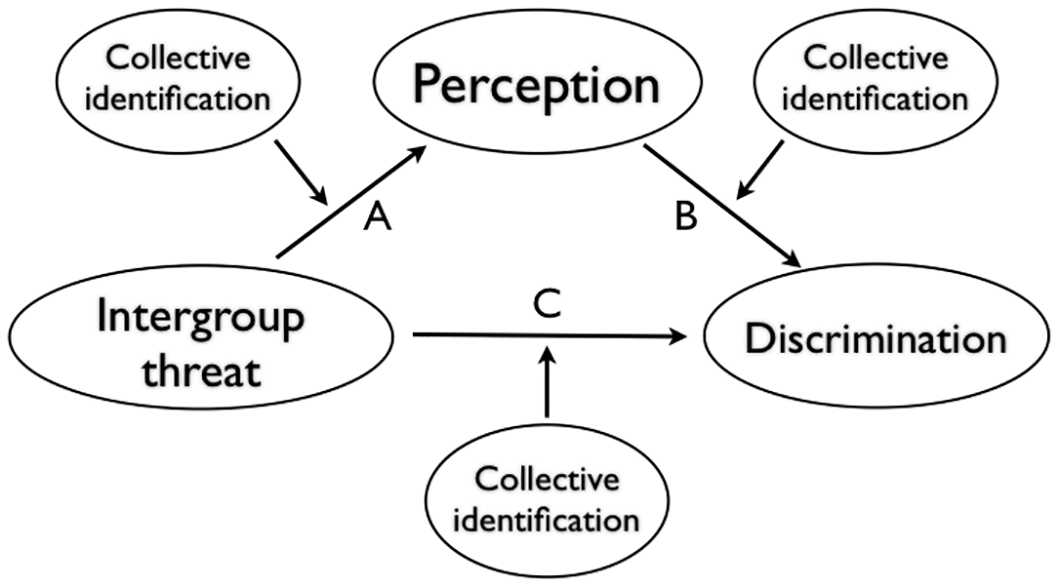
Proximity Under Threat. According to our working framework, intergroup threat biases estimation of distance (making threatening out-group members seem closer), which can increase discrimination. All of these factors are subject to the degree to which people identify with their in-group.

Indeed, according to the Perceptual Model of Intergroup Relations [26], perception can serve as a psychological process underlying the attitudinal and behavioral consequences of social identities. Here, we argue that distance judgment is a special case under this broader model. Specifically, we propose that it is often functionally adaptive for people to estimate that a threatening out-group is physically closer than a less threatening group (see Fig 1, path A) in order to facilitate preparation for action against this out-group (see Fig 1, path B). Physical proximity to a threatening out-group often places one in danger of losing status, resources, or worse. Therefore, physical proximity between competing groups should be associated with heightened animosity and a desire to remove the out-group from the in-group’s physical space. Thus, we also predict that intergroup threat might increase estimates of physical proximity, which could promote action to keep the out-group at a distance, such as the construction of barriers.

Importantly, the strengths of each of the relationships should differ as a function of in-group identification. Reactions to experiencing intergroup threat should depend not only on the type of threat, but also the psychological significance of a particular collective identity—the degree of importance of a particular group membership to the individual’s overall self-concept [7, 27, 28]. For instance, a negative comparison with an out-group can lead high-identifiers to engage in group-level defensive reactions, without having an impact on low-identifiers [4]. In general, high identifiers should be more sensitive to the influence of intergroup threat on perceptual judgments [7]. High identifiers may also be sensitive to other features of the intergroup situation, such as the presence of in-group support [25]. Taken together, it would appear that intergroup threat influences distance judgments among highly identified group members.

In the current research, we assessed both physical and psychological factors that can flexibly alter the effects of intergroup threat on distance judgment and discrimination. Specifically, we examined three novel ways to alleviate the negative intergroup consequences of threat-induced proximity as well as the downstream consequences of physical proximity on intergroup relations. First, we assessed whether fulfilling the *security needs* of the threatened in-group facilitates positive intergroup relations by eliminating the threat-induced proximity effect. Second, we examined the utility of *intergroup apology* to eliminate biased distance judgment as a result of out-group transgression. Third, we *manipulated perceived proximity* of the threatening out-group to assess whether representing a threatening out-group as too close to one’s own group can enhance support for discriminatory policies toward the out-group.

#### Distance judgment meets security needs

To understand why distance judgment might be altered in the face of intergroup threat, it is valuable to consider the needs of the group under threat [29, 30]. According to Bar-Tal [31], the ethos of intergroup conflict cannot be properly understood without taking into account the security concerns of both groups [32]. Specifically, beliefs about in-group security drive both perception and behavior in the name of contemporary safety and future vitality [33]. As such, we hypothesized that physical barriers between the in-group and a threatening out-group might reduce the threat-induced proximity effect.

Throughout history, intergroup barriers have been used to keep out threatening intruders. Groups have erected physical barriers as a means of protection: The great wall was built by China to protect them from Mongolian invaders, the Berlin Wall was built by East Germany to protect again the influence of the West, and the Separation Wall was built by Israel to protect against Palestinian attacks. In each case, the in-group believed the out-group threat was imminent to justify investing resources to create a physical barrier. A strong intergroup barrier is not just a physical boundary between groups, but may also serve a protective psychological function. We reasoned that these barriers allow people to feel that they have successfully “pushed away” the enemy—helping them obtain a sense of security and reducing perceived proximity. We tested whether a strong (versus weak) barrier between the in-group and a threatening out-group could alter the relationship between feelings of threat and representation of physical reality. We reasoned that a secure intergroup barrier might effectively weaken the relationship between perceived intergroup threat and distance judgment, insofar as the barrier provides a protective function against threat.

#### The function of intergroup apology

Building a wall between large groups is a costly and contentious activity. A significantly less costly, and more socially constructive, option for reducing threat-induced proximity is apology. A vast literature suggests an interpersonal apology reduces the desire for retribution and increase forgiveness [34–36]. However, the link between intergroup apology and forgiveness is more tenuous [37, 38]. Whereas some empirical research has shown that intergroup apology yields forgiveness [39, 40], other research has found that intergroup apology yields little forgiveness [41]. The weakness of the intergroup apology-forgiveness link may be due, in part, to intergroup friendships.

It has been long established that contact, particularly high quality cross-group friendship, promotes positive intergroup relations [2, 42]. However, the perceived severity of a transgression may be worse when victims have cross-group friends. This is because it may be especially painful to be harmed by those considered a friend. Dante reserved the lowest circle of hell for betrayal. Research confirms that harm originating from close others heightens a sense of betrayal, which undermines forgiveness [43].These feelings of betrayal should be absent when harm is done by out-group members who are not friends. In this light, an intergroup apology may reduce the threat-induced proximity effect among people who lack pre-existing intergroup friendships. This would not only establish the role of security in distance judgment, but introduce a more pro-social means for reducing intergroup conflict than erecting an expensive barrier.

## Overview of Current Studies

We examined whether representation of physical reality are strengthened and weakened by intergroup constructs, and that these representations have important consequences. Specifically, this paper presents three studies designed to (1) provide evidence that judgments of physical reality are shaped by social identity and intergroup threat, (2) examine psychological factors that can reduce the effects of threat on distance judgment, and (3) to establish the attitudinal and behavioral consequences of altered distance. We examined multiple social groups with different intergroup dynamics to help establish the generalizability of our proposed framework for intergroup contexts.

We also assessed the consequence of manipulating intergroup distance. People are motivated to maintain positive feelings about their in-group [3, 44], especially in the presence of threats from out-groups [4]. This motivation may manifest in various behavioral outcomes, including in-group favoritism, out-group derogation, self-stereotyping, and/or other forms of collective resistance [27], depending on the psychological significance of a particular collective identity [28]. As such, altered distance judgments could have important consequences for intergroup relations. We hypothesized that out-group derogation would be enhanced when the threatening group was made perceptually close, and minimized when the threatening group was perceptually far.

## Study 1: Good Fences Make Good Neighbors

Good fences make good neighbors.

-Robert Frost, *Mending Wall*

Intergroup barriers serve as a physical divide between political and military entities, but they may also serve a psychological function for members of bordering groups [45]. In Study 1, we explored the role of a strong intergroup barrier in eliminating threat-induced physical closeness (see Fig 1, moderator of path A). In previous research, Americans who reported higher levels of symbolic threat from Mexican immigration estimated Mexico City to be closer [7]. Mexican immigrants are the biggest immigrant group in the US, with a 17-fold increase since 1970 [46]. As such, managing Mexican immigration is a source of considerable debate in the US. To better understand this issue, we manipulated the perceived strength of an intergroup barrier—in this case the US-Mexico border—and assessed how it moderated the relationship between threat and distance judgment. We hypothesized that when the intergroup barrier was perceived as weak, feelings of intergroup threat would be associated with intergroup proximity. However, this relationship should be eliminated (or even reversed) when an intergroup barrier was strong.

We also examined the effects of intergroup threat on other dimensions of physical reality. Biases in perceived proximity often co-occur with perceptual biases along other dimensions, such as size. Threatening stimuli appear larger than neutral or positive stimuli [47], which may be beneficial for survival because exaggerating the size of threatening objects may make them easier to detect in the environment and avoided [24]. In the context of *intergroup* threat, we operationalized the size of a group as the number of group members. More than half a century ago, Allport and Kramer [48] found that students high on anti-Semitic prejudice (compared to those low in anti-Semitic prejudice) who were shown an array of photographs judged more of the targets to be Jewish. Moreover, members of many majority groups often overestimate the population of minority groups and the rate at which minority groups are growing, and this overestimation predicts attitudes towards immigrants and minority groups [49–51]. Similarly, perceived (but not actual) size of the foreigner population in Germany predicted Germans’ feelings of threat and discriminatory attitudes toward foreigners in their country [52]. All these findings suggest that threatening groups may be perceived to be closer *and* larger. Therefore, we reasoned that threat may promote not only physical closeness of the threatening stimuli, but also exaggerated size judgment. We predicted parallel effects to our distance effects, such that higher perceived threat from Mexican immigration would predict larger group size judgment when there was a weak intergroup barrier. However, this effect would be eliminated when there was a strong intergroup barrier

### Method

#### Ethical issues

The research reported in this paper involves human subjects, and has been approved by the New York University Committee on Activities Involving Human Subjects (Studies 1 & 3; approval #10–7878), and the Carleton University Psychology Research Ethics Board (Study 2; approval #12–156). Participants provided written informed consent to participate in this research, and the ethics committees approved this consent procedure.

#### Participants

We recruited an online sample of 101 American participants (Mean age = 33.4, *SD* = 12.5; 46% male) in March 2012 through Amazon’s Mechanical Turk to participate in the current study (79% White/Caucasian, 10% African American, 4% Hispanic, 5% Asian, and 2% other). Recent research evaluating Amazon’s MTurk as a source for psychological research subjects has shown that MTurk provides a reliable and diverse subject pool that behaves in ways consistent with known effects in psychology [53, 54]. Of the 101 participants in this study, six participants (6% of all participants) failed our attention check and were therefore excluded from our analyses. All following analyses were done using the data of 95 participants (Mean age = 33.4, *SD* = 12.5; 46% male). Each participant was compensated $0.30 for online participation. All of our participants reported to be currently living in the US. This is important because we ask participants to make their distance estimations from their current location in the US. Based on previous research [7], we determined all sample sizes based on a small to medium effect size and .80 power.

#### Procedure

Participants read a consent form and agreed to participate in this research and contribute their responses. We first asked participants to indicate their current city of residence, so that we could calibrate each participant’s distance estimation (measured later) to actual distance. Then participants reported their level of perceived symbolic threat from Mexican immigration. We used the 12-item perceived symbolic threat scale modified to specifically refer to Mexican immigration (e.g., “Mexican immigration is undermining American culture.” [55]. Participants indicated the extent to which they agreed or disagreed with each statement on a 7-point scale (-3 = *strongly disagree*, 0 = *neutral*, 3 = *strongly agree*).

Following the symbolic threat assessment, we manipulated the strength and security of the US-Mexico border, by having participants read one of two versions of a news article allegedly from CNN.com. The articles were created to maximize ecological validity. Half of our participants were randomly assigned to the strong border condition, in which the news article depicted the US-Mexico border as “among the most heavily protected in the world”, “bringing Mexican immigration down by a significant amount”, whereas the other half of the participants in the weak border condition read the article depicting the border as “one of the most frequently crossed”, “largely unsecured”, “the effectiveness of which is highly questionable” etc. As a manipulation check, we asked participants to rate the effectiveness of the US-Mexico border in keeping out illegal immigration immediately after the article on a 7-point scale (-3 = *very ineffective*, 0 = *neither effective nor ineffective*, 3 = *very effective*; *M* = -.65, *SD* = 1.83).

Participants then estimated the distance in a straight line from their current location to Mexico City, Mexico; Los Angeles, USA; and Vancouver, Canada. We included Los Angeles as a domestic city and Vancouver, Canada as a non-threatening foreign city (see Study 2 in [7]). Participants were instructed to estimate these distances in a straight line by marking on a line representing five thousand miles. We have used this measure of distance estimation in previous research [7]. Then we had participants estimate the size of the Mexican immigrant population in the US. Specifically, participants made their estimation on a scale from zero to 20 million (actual = approximately 13 million). Lastly we collected demographic information, including age, gender, and race. All participants were thanked for their participation and were given the opportunity to leave any comment they may have. The experimental materials, data, and analyses scripts from all studies reported in this paper are available from the Open Science Framework website (accession number osf.io/k8mtj/).

#### Statistical analyses

We first computed a symbolic threat score for each participant. Half of the items on the symbolic threat scale were reverse worded and recoded such that higher scores indicate stronger perceived threat. Our 12-item symbolic threat scale was highly reliable (α = .90). We created a composite symbolic threat score for each participant by averaging the 12 items (*M* = .09; *SD* = 1.16).

We then computed a distance estimation ratio for each participant calibrated based on his or her respective location (ratio = estimated/actual distance). For a participant living in Portland, Oregon, for example, the actual distance between Portland, Oregon and Mexico City, Mexico is 2092 miles. If this participant made an estimation of 1450 miles, then his/her ratio for distance estimation to Mexico City would be 1450/2092 = 0.69. We calculated this ratio for distance estimation to Mexico City, and the two control cities (LA and Vancouver). Similarly, we computed a size estimation ratio (ratio = estimated/actual) for each participant.

We first conducted preliminary analyses to check the effectiveness of our strong versus weak border manipulation. We conducted a one-way analysis of variance (ANOVA) to compare responses on the manipulation check for participants in the strong versus weak border conditions. Then we conducted regression analyses on the distance estimation ratio and size estimation ratio. Specifically, we regressed both dependent variables on the mean-centered perceived symbolic threat score, border strength condition (weak border = 0; strong border = 1), and the interaction term [56].

### Results

#### Manipulation check

Our strong versus weak border manipulation check indicated that participants in the strong border condition evaluated the US-Mexico border to be stronger (*M* = 0.69, *SD* = 1.24) compared to those in the weak border condition (*M* = -2.02, *SD* = 1.21), *F*(1, 93) = 116.34, *p* < .001, η^2^ = .56, indicating that our manipulation was effective.

#### Distance judgment

To examine whether our border strength manipulation had an effect on estimated distance to Mexico City as a function of participants’ perceived threat from Mexican immigration, we regressed estimated distance to Mexico City (ratio) on mean-centered perceived symbolic threat, border strength condition (weak border = 0; strong border = 1), and the interaction term, *R*^*2*^ = .09, *F*(3, 91) = 2.80, *p* = .04. Replicating our previous research [7], symbolic threat predicted estimated distance to Mexico City, *t*(91) = -1.88, *p* = .06, *B* = -.19, 95% CI [-.39, .01], such that greater perceived symbolic threat from Mexican immigrants was associated with marginally *shorter* estimated distance to Mexico City.

**Fig 2:**
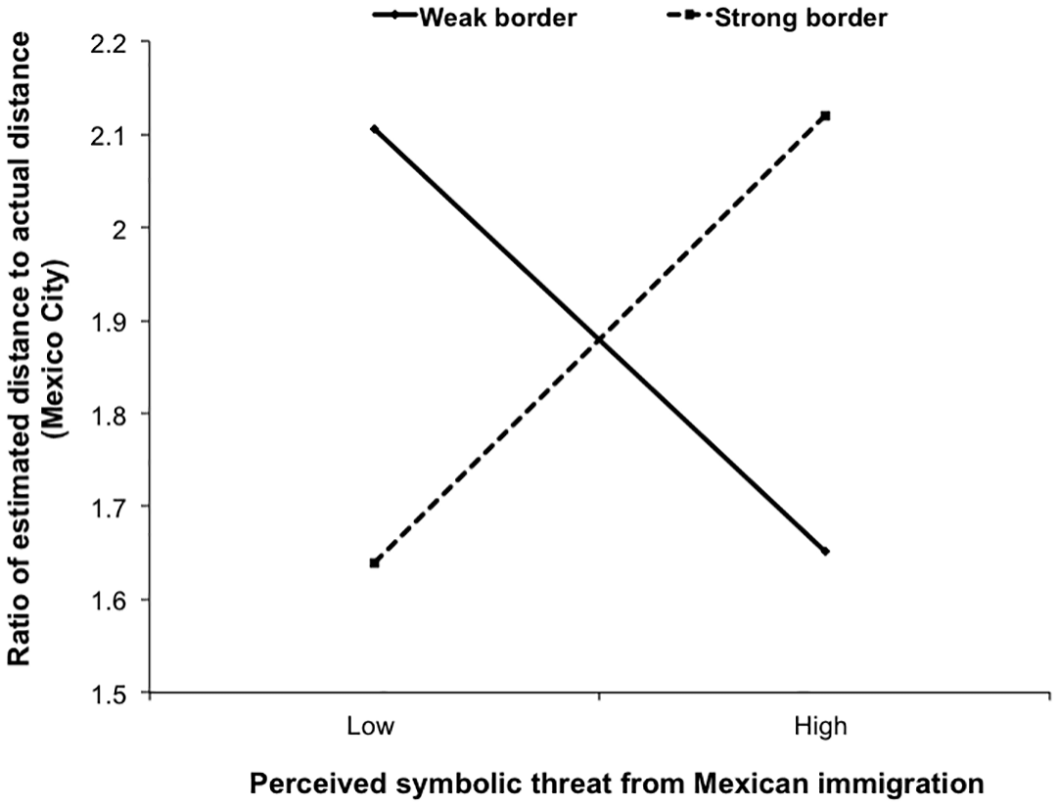
Distance estimates as a function of perceived threat and US-Mexico border strength manipulation.

More importantly, this relationship was moderated by our border strength manipulation. The interaction between perceived symbolic threat from Mexican immigration and the border strength manipulation was significantly associated with estimated distance to Mexico City, *t*(91) = 2.87, *p* = .005, *B* = .39, 95% CI [.12, .66] (see Fig 2). Replicated our previous findings, in the *weak* border condition stronger symbolic threat from Mexican immigration predicted closer estimated distance to Mexico City, *t*(91) = -2.44, *p* = .02, *B* = -.38, 95% CI [-.69, -.07]. However, in the *strong* border condition, this relationship was eliminated, *t*(91) = .08, *p* = .94, *B* = .01, 95% CI [-.12, .13]. Although we report simple slopes analyses using the overall error terms, conducting simple slopes analyses by splitting the data by the categorical variable—border strength manipulation [56] does not change overall the findings of this research, confirming that the findings are robust to analytic strategy. This also applied to simple slopes analyses reported in Studies 2 and 3. Therefore, a secure intergroup barrier eliminated the estimated proximity induced by intergroup threat.

None of the distance estimation effects reported above emerged for distance estimation to the two control cities: LA and Vancouver (*p*s > .20), suggesting that the effects of perceived symbolic threat on distance estimation as a function of border strength were specific to the group posing the relevant threat, and not to other non-threatening out-groups. Thus, all these effects of threat were specific to Mexico City.

#### Population size judgment

**Fig 3:**
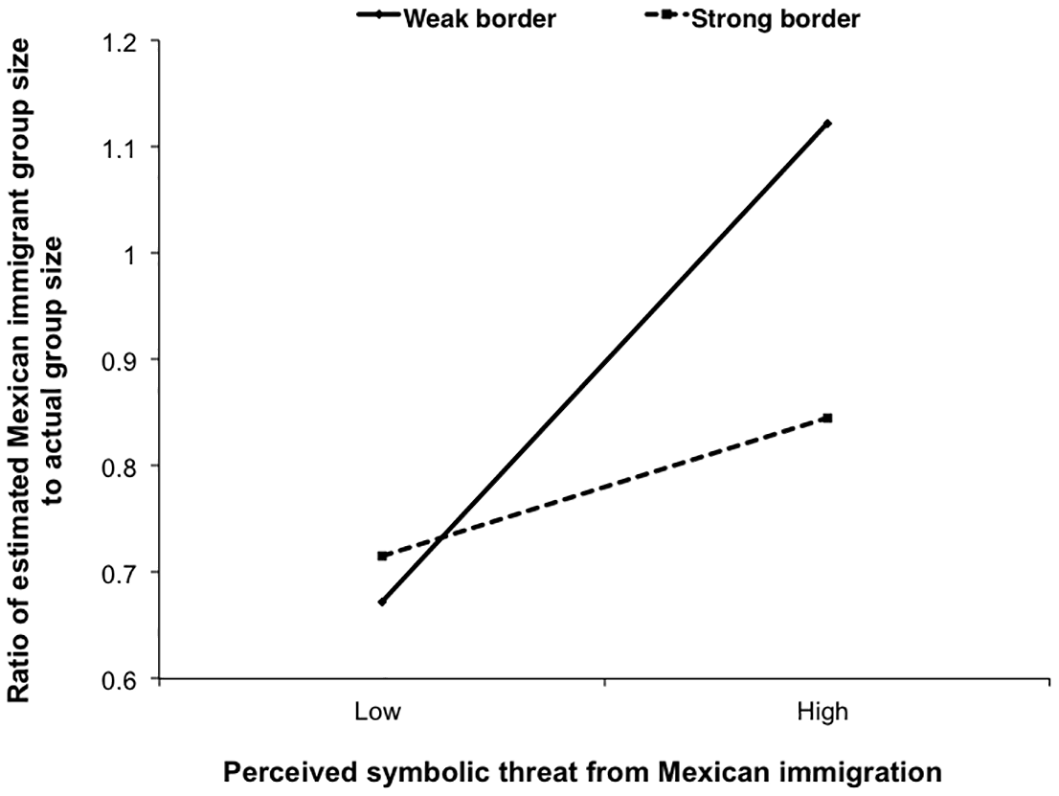
Size estimates as a function of perceived threat and US-Mexico border strength manipulation.

We regressed size estimation (ratio = estimated/actual) on mean-centered perceived symbolic threat, border strength condition (weak border = 0; strong border = 1), and the interaction term, *R*^*2*^ = .16, *F*(3, 91) = 5.90, *p* = .001. Replicating previous findings with distance estimation, symbolic threat predicted population estimates, *t*(91) = 3.80, *p* < .001, *B* = .19, 95% CI [.09, .28], such that greater perceived symbolic threat from Mexican immigrants was associated with *larger* population estimates. This relationship was moderated by the border strength condition, *t*(91) = -2.00, *p* = .05, *B* = -.13, 95% CI [-.26, -.00] (see Fig 3). Simple slopes analysis indicated that in the weak border condition, stronger symbolic threat from Mexican immigration predicted larger population estimates, *t*(91) = 3.64, *p <* .001, *B* = .12, 95% CI [.05, .19]. However, in the strong border condition, this association was in the opposite direction, *t*(91) = 3.29, *p* = .001, *B* = .25, 95% CI [.10, .41]. Parallel to the distance estimation effects, a strong intergroup barrier eliminated or reversed the exaggerated size judgment of a threatening out-group.

### Discussion

Findings from this study suggest that intergroup barriers may serve an important psychological function. When intergroup barriers were weak, intergroup threat was associated with exaggerated proximity to the threatening out-group. However, this effect was reduced when there was a strong and secure barrier. Importantly, none of these findings emerged for either control city, indicating that these effects were specific to a threatening out-group. Thus, constructing barriers between groups may have a palliative effect for threatened individuals, reducing their feelings of threat and biased distance and size judgments.

Our previous research served as initial evidence of our proposal about the effect of intergroup threat on judgment of physical properties associated with the threatening group [7]. The contributions of the current study were two-fold. First, we demonstrated that the influence of social identity and intergroup threat on judgment of physical reality was complex—beliefs about the strength of an intergroup barrier can moderate this relationship. Second, we found that the effects of intergroup threat extend beyond distance judgments to other aspects of physical reality. This study provides support for the flexibility of the relationship between intergroup threat and physical distance (Fig 1, path A). In the next study, we sought to bolster our argument by testing this path with an alternative strategy for reducing intergroup threat—forgiveness.

## Study 2: Apology, Forgiveness, and Distance

Study 1 provided initial evidence that a secure physical barrier between groups can reduce estimated proximity to a threatening out-group. However, building barriers is an expensive and contentious way to promote in-group security. In Study 2, we proposed and tested a more economical and socially harmonious way in which the perceived physical proximity can be reduced—intergroup apology. In this study, we heightened intergroup threat by informing in-group members that the out-group derogated the value of the in-group [57]. We hypothesized that this threat would elicit the proximity under threat effect (see Fig 1, moderator of path A). We then examined whether the presence of an intergroup apology would reduce estimates of proximity.

Past research has found mixed evidence regarding whether intergroup apology yields pro-social outcomes. For example, some research has found that an intergroup apology can facilitate intergroup forgiveness [39, 40], while other research found no such effect of intergroup forgiveness [41]. Importantly, the utility of an intergroup apology to yield intergroup forgiveness is limited by the severity of the harm [37]. More severe forms of harm make it more difficult for an apology to elicit forgiveness. One of the most severe offenses someone can commit is betrayal [58], and group members may be less forgiving when they feel betrayed [43].

In addition to examining the role of intergroup apology on judgments of physical distance, we also explored how cross-group friendships affect the relationship between apology and physical distance judgment. Contact between people of different groups normally helps promote positive intergroup relations [2]. However, not all forms of contact are equally beneficial in improving attitudes, judgments, and behaviors [2, 42, 59]. Within the context of an intergroup transgression, victimized group members who have close friendships with members of the perpetrator group prior to the transgression often feel betrayal. Conversely, people who have no or low quality cross-group friendships are unlikely to experience the transgression as betrayal. Consequently, people with no or low quality cross-group friendships are more likely to feel a greater sense of security when an apology is offered by the out-group, which should manifest in a reduced threat-induce proximity effect. Alternatively, cross-group friendships might enhance the effects of intergroup apology. We directly tested these competing predictions.

### Methods

#### Participants

Seventy-one Carleton University students participated in this study in the Fall 2012 semester, and we stopped data collection when the semester ended. Seven participants did not consent to their data being used for research purposes and two had incomplete data. As such, a total of 64 participants (Mean age = 19.88, *SD* = 4.00; 26% males) were included in analyses. Participants received partial course credits for their participation.

#### Procedure

Participants first completed measures to assess their collective identification with the in-group (Carleton University) and their friendships with out-group (University of Ottawa) members. We chose University of Ottawa as the potentially threatening out-group to Carleton University students because University of Ottawa is considered the more prestigious of the two universities located in Ottawa [60] and the two universities regularly compete for students and in university athletics events.

To ensure that existing friendships with out-group members did not affect the extent to which participants identified with their in-group, participants completed a 4-item collective identification scale (α = .77) including items such as “I identify with Carleton University.” Participants indicated the extent to which they agreed or disagreed with each statement on a 5-point Likert-type scale (from 1 = *strongly disagree* to 5 = *strongly agree*).

To measure quantity of contact with the out-group, participants were asked to indicate the number of friends they had who attended the University of Ottawa. To assess quality of cross-group friendship, they indicated on a 5-point Likert-type scale (from 1 = *strongly disagree* to 5 = *strongly agree*) the extent to which they agreed with the statements “I feel close to my friends who attend the University of Ottawa” and “My friends who attend the university of Ottawa are similar to me” [61, 62].

Then we experimentally manipulated intergroup apology. Participants were asked to read an ostensibly real newspaper article published by Carleton University. This article described comments from an Ottawa newspaper about a derogatory remark made by the president of the University of Ottawa concerning the academic reputation of Carleton University. It was explained that the story was posted on the University of Ottawa’s Twitter and Facebook accounts and it was reported that these posts received over a thousand ‘likes’ and approving comments from University of Ottawa students. Importantly, the story varied in one key respect: the presence or absence of an apology. Half of the participants were randomly assigned to read an additional paragraph detailing an apology made by the President of the University of Ottawa for the transgression. The other half of the participants read that the President of the University of Ottawa had yet to take responsibility or offer an apology. Following the news article, we gave participants a manipulation check to ensure that those in the apology condition were aware that an apology was offered and that those in the no apology condition were aware that an apology was not yet offered. This was assessed with a multiple choice question where participants were to respond by checking “yes” or “no” to the question “according to the article, the University of Ottawa has apologized”.

Then forgiveness was assessed using the Transgression-Related Interpersonal Motivations (TRIM) [34] scale, which assesses motives of revenge and avoidance (α = .93). Participants were asked to what extent they agreed or disagreed with statements on a 5-point Likert-type scale (from 1 = *“strongly disagree”* to 5 = *“strongly agree”*). For ease of interpretation, TRIM scores were reverse coded such that higher values reflect higher degree of forgiveness.

Participants then estimated the physical distance between Carleton University (the in-group) and the University of Ottawa (the out-group) using a line measure. The line was anchored at “as physically close as it can possibly be” and “as physically distant as it can possibly be”, and required participants to indicate on the line how close or distant the University of Ottawa felt to them. The two universities are both located in Ottawa, Ontario, Canada, and are approximately 3.7 miles driving distance apart.

The final part of the questionnaire contained demographic questions asking the participants to indicate their age, sex, and year of study. Upon completion of the survey, or withdrawal from the study, a debriefing form was presented to participants on the survey hosting website. Following the debriefing, participants were asked whether they consented to their data being used for research purposes.

#### Statistical analyses

We computed a composite collective identification score for each participant. Two of the four items on this scale were reverse-coded. The composite collective identification score was computed by summing the four items for each participant (*M* = 17.36; *SD* = 2.42). We then computed a composite measure of cross-group friendship quality by summing the two quality of cross-group friendship items (*r* = .75; *M* = 5.00, *SD* = 3.58). A forgiveness score was computed for each participant by averaging responses on the Transgression-Related Interpersonal Motivations (TRIM) scale (*M* = 5.76, *SD* = .78).

We first conducted a manipulation check to determine whether participants correctly encoded the forgiveness manipulation. We then performed regression analyses on the forgiveness variable and the distance estimation variable. We regressed both dependent variables on the apology manipulation variable (no apology = 0; apology = 1), the mean-centered quality of cross-group friendships variable, and the interaction term [56]. Finally we regressed both dependent variables on the apology manipulation variable (no apology = 0; apology = 1), the quantity of cross-group friends variable, and the interaction term.

### Results

#### Manipulation check

Of the 64 participants in this study, 10 participants (16% of all participants) failed to correctly identify the forgiveness manipulation and were therefore excluded from analyses. The following analyses were done using data from 54 participants (*Mean age* = 19.93, *SD* = 4.03; 22% males).

#### Forgiveness

To examine how out-group friendships affected feelings of forgiveness following an apology, we regressed forgiveness on the apology manipulation variable (no apology = 0; apology = 1), the mean-centered quality of cross-group friendships variable, and the interaction term, *R*^*2*^ = .23, *F*(3, 44) = 4.42, *p* = .008. Only quality of out-group friendship significantly predicted forgiveness, *t*(44) = 3.56, *p* = .001, *B* = .40, 95% CI [.17, .63], such that higher quality of out-group friendship was associated with greater forgiveness. We conducted parallel analyses with quantity of cross-group friendships, *F*(3, 50) = .41, *p* = .74, *R*^*2*^ = .02, and no statistically significant effect emerged.

#### Distance judgment

**Fig 4:**
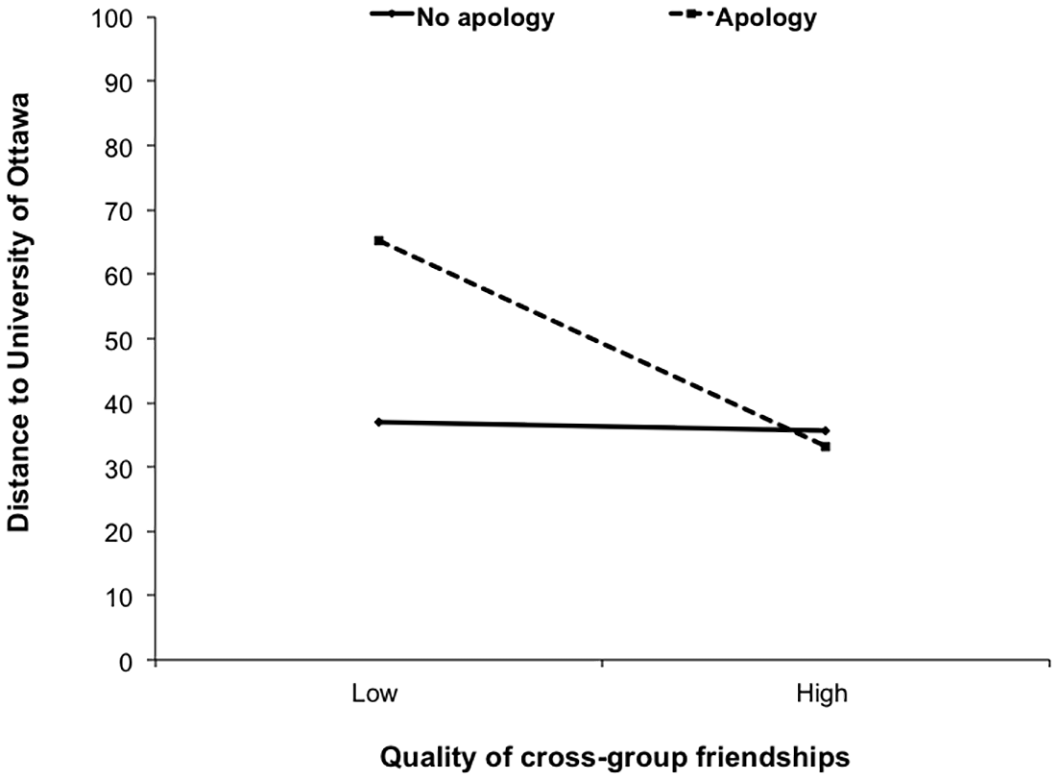
Perceived physical distance to the out-group estimated by participants who reported the quality of their cross-group friendships with out-group members, as a function of whether or not they were presented with an apology from the out-group.

To assess the role of quality of cross group friendships on the estimated distance from the out-group, subjective distance estimation scores were regressed on the apology manipulation variable (no apology = 0, apology = 1), the mean-centered quality of cross-group friendships variable, and the interaction term, *F*(3, 43) = 3.92, *p* = .02, *R*^*2*^ = .22. As predicted, participants who received an apology estimated that the out-group was physically farther away, compared to those who did not receive an apology, *t*(43) = 2.23, *p* = .03, *B* = 12.88, 95% CI [1.22, 24.53]. This was qualified by a significant interaction between the apology manipulation by the quality of out-group friendships, *t*(43) = -2.37, *p* = .02, *B* = -14.31, 95% CI [-26.46, -2.14]. The apology manipulation increased estimated subjective distance for people with low quality out-group friendships, *t*(43) = 3.13, *p* = .01, *B* = 28.16, 95% CI [10.02, 46.29], but not for those with high quality out-group friendships, *t*(43) = -.29, *p* = .77, *B* = -2.40, 95% CI [-19.14, 14.33] (see Fig 4). In other words, apology reduced estimated proximity, particularly among those with low quality out-group friendships.

To assess the role that the number of cross-group friendships plays in perceived distance from the out-group, we conducted parallel analyses with quantity of out-group friendships, *R*^*2*^ = .10, *F*(3, 49) = 1.90, *p* = .14. None of the effects reported above emerged for quantity of cross-group friendships. Importantly, we also conducted correlation analyses to make sure that neither quality of cross-group friendships nor number of cross-group friends was correlated with collective identification with Carleton University, *p* = .43 and *p* = .35 respectively. Therefore, effects of quality of cross-group friendships could not simply be attributed to variations in students’ collective in-group identification.

### Discussion

The goal of the study was to investigate whether an apology from an out-group following a transgression would alter the victimized group’s judgment of physical properties associated with the out-group. This study tested the extent to which the relationship between intergroup threat and physical distance—as shown in previous research—is prone to influence from intergroup dynamics. The results suggest that people with high quality cross-group friends may feel betrayed by hurtful comments by the threatening out-group, and thus an apology may do relatively little to reduce their feelings of threat. Conversely, for in-group members with low quality cross-group friendships, an apology may reduce feelings of threat and manifest in a reduced effects of threat on distance estimates. Results were largely consistent with these hypotheses.

Given the tenuous relationship between intergroup apology and forgiveness [37], it is not surprising that the presence of an apology did not influence intergroup forgiveness—there are several factors that can undermine the difficulty to traverse intergroup forgiveness process [33]. Nevertheless, this experiment suggests that although an intergroup apology did not influence the emotionally laden process of forgiveness, it can alter distance estimates among people who are not apt to feel betrayed by a wrongdoing. Together, findings from Studies 1 and 2 suggest that intergroup threat leads to proximity in physical distance judgments, but this relationship is sensitive to factors that may change the intergroup dynamics.

## Study 3: When Closeness Breeds Contempt

“Distance has the same effect on the mind as on the eye.”

- Samuel Johnson, *The History of Rasselas*, *Prince of Abissinia*

In line with Johnsons’ statement, we hypothesized that distance judgment may impact the group member’s *mind*, especially their attitudes and intentions towards members of threatening out-groups. In Study 3, we directly manipulated the proposed mediator—physical proximity—and examined the effects on intergroup discrimination. We measured group members’ discriminatory attitudes against a threatening out-group *and* a seating chart measure to assess behavioral intentions towards members of this out-group. We hypothesized that when a threatening out-group appeared physically close, highly identified members of the threatened group would express more support for discriminatory policies *and* choose to be physically farther away from members of the threatening out-group. Study 3 provided a critical test of a critical component of our proposed framework (Fig 1: Path B)—the downstream consequences of physical proximity.

We tested our hypotheses in the context of an intense intergroup rivalry between two professional baseball teams: the New York Yankees and the Boston Red Sox. The Yankees and the Red Sox have been rivals in Major League Baseball for the past century. Indeed, their rivalry is arguably the fiercest in North American professional sports [63]. Although the Yankees (27) have historically won many more championships than the Red Sox (8), the Red Sox have improved significantly in the past decade, posing a major threat to the Yankees. Previous research has capitalized on the fierce intergroup rivalry and threat, and shown that fans of both teams are more likely to display aggression towards a rival Red Sox or Yankees fan, respectively, than towards a non-rival Orioles fan [64].

### Method

#### Participants

Two hundred and eighty-seven Yankees fans participated in our study online through Amazon’s Mechanical Turk in October 2011. We recruited more participants than indicated by our a priori power analysis for this experiment because 1) in our research we find that exclusion of participants based on attention checks is usually much higher than laboratory studies; and 2) we anticipated to exclude even more participants based on checks of Yankees fan identity. Of the 287 participants, 51 participants (18%) failed our attention check and were therefore excluded from analyses. The following analyses were done using the data from 236 participants (mean age = 32.1; *SD* = 12.2; 69% male). Each participant received $0.15 in compensation for online participation. All of our participants reported that they were currently living in the US.

#### Procedure

Participants were randomly assigned to one of two distance manipulation conditions: close versus far. As our physical distance manipulation, participants saw a Cartesian grid with logos of the two teams. To engage participants with this manipulation, we asked participants to click on the logo of their favorite team. In this case, all participants clicked on the Yankees logo. In the *close* condition, the two logos were placed right next to each other, whereas in the *far* condition, they were placed far apart. This subtle manipulation has been used in previous research and proved to be an effective manipulation of perception of physical distance [65].

Participants first completed a measure of their collective in-group identification. We used a 3-item modified version of the collective identification scale [66], which included items such as “I am proud to be a Yankees fan.” Participants indicated the extent to which they agreed or disagreed with each statement on a 7-point scale (-3 = *strongly disagree*, 0 = *neutral*, 3 = *strongly agree*). Then we assessed discriminatory attitudes by measuring support for policies favoring Yankees over Red Sox fans. We measured participants’ attitudes towards six policies that favored one team over the other, which included items such as “Yankee fans should have priority over Red Sox fans for seat selection”. Participants indicated the extent to which they agreed or disagreed with each statement on a 7-point scale (-3 = *strongly disagree*, 0 = *neutral*, 3 = *strongly agree*).

In addition to discriminatory attitudes, we also assessed behavioral intention towards out-group members in terms of how far Yankees fans voluntarily choose to sit from Red Sox fans at a game. We first showed participants a Yankee Stadium seating chart and asked them to indicate where *they* would mostly likely sit if they were going to a Yankees-versus-Red Sox game. Then on a separate page they saw another Yankee Stadium seating chart and indicated where they would like to have *Red Sox fans* sit in the stadium at the Yankees-versus-Red Sox game. Lastly, we collected demographic information including age and gender.

#### Statistical analyses

We computed a composite collective identification score for each participant. One of the three items was reverse-coded. Our 3-item collective Yankees identification was moderately reliable (α = .66). The composite collective identification score was computed by summing the three items (*M* = 0.23; *SD* = 3.34). Then we computed a composite discriminatory attitudes score for each participant. Half of the items were reverse-coded. Our 6-item discriminatory policy measure was reliable (α = .70). The composite score was created by averaging across the six items (*M* = 1.83, *SD* = 0.95). Finally, we computed an intergroup seating distance index for each participant by measuring, for each participant, the physical distance between his/her own chosen seat and preferred seat for Red Sox fans on the seating charts.

To examine whether our distance manipulation altered participants’ discriminatory attitudes and behavioral intentions as a function of their collective identification with the Yankees, we conducted regression analyses on the discriminatory attitude score and the seating distance measure. Specifically, we regressed both variables on mean-centered collective identification with the Yankees, distance condition (far = 0; close = 1), and the interaction term [56].

### Results

#### Discriminatory attitudes

We regressed the discriminatory attitude score on mean-centered collective identification with the Yankees, distance condition (far = 0; close = 1), and the interaction term, *F*(3, 232) = 18.85, *p* < .001, *R*^*2*^ = .20. Replicating previous research [4, 28], collective identification with the Yankees predicted discriminatory attitudes towards the Red Sox, *t*(232) = 3.22, *p* = .001, *B* = .74, 95% CI [.29, 1.19]. Specifically, collective identification with the in-group (Yankees) was positively associated with greater discriminatory attitudes towards the threatening out-group (Red Sox).

**Fig 5:**
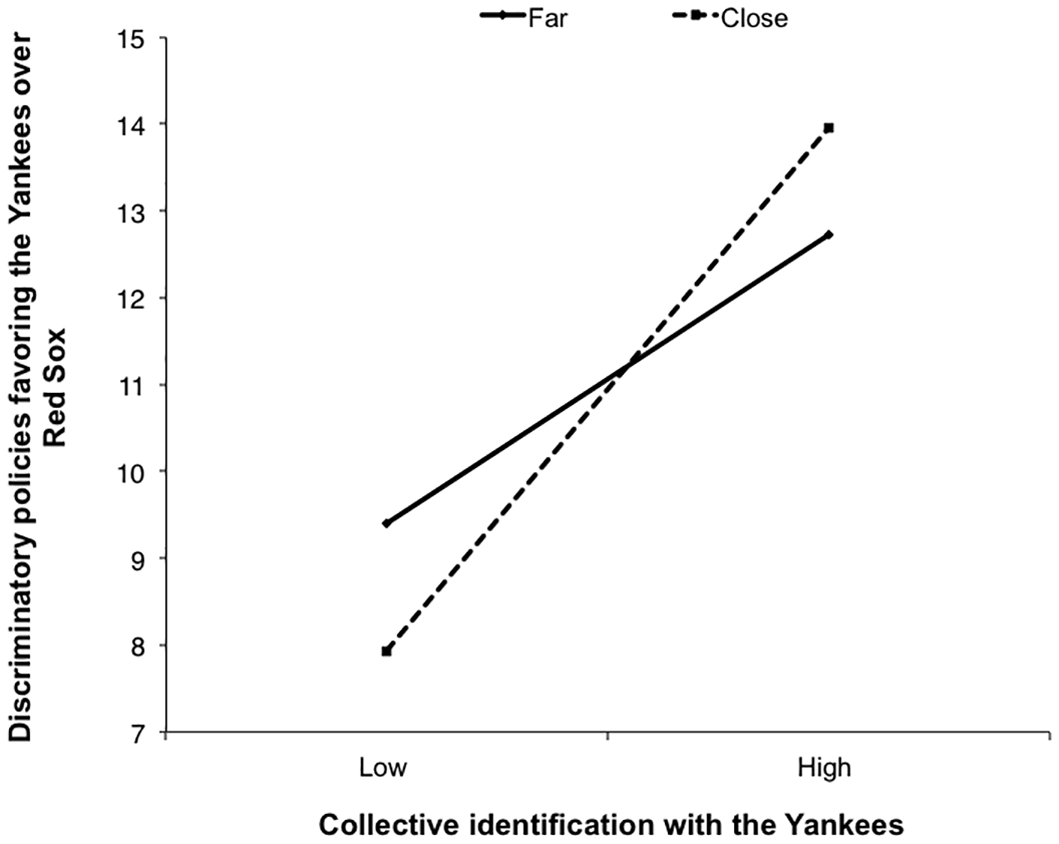
Support for discriminatory policies as a function of collective identification and distance manipulation.

More importantly, this relationship was moderated by our distance condition. The interaction between collective identification and the distance condition was significantly associated with discriminatory attitudes, *t*(232) = 1.98, *p* = .05, *B* = .60, 95% CI [.00, 1.19] (see Fig 5). Simple slopes analysis indicated that collective identification predicted discriminatory attitudes in the close condition, *t*(232) = 6.86, *p* < .001, *B* = 1.03, 95% CI [.74, 1.33]; but not in the far condition, *t*(232) = 1.23, *p* = .22, *B* = .44, 95% CI [-.27, 1.14]. In other words, physical proximity increased the relationship between collective identification and support for out-group discrimination.

#### Intergroup seating distance

**Fig 6:**
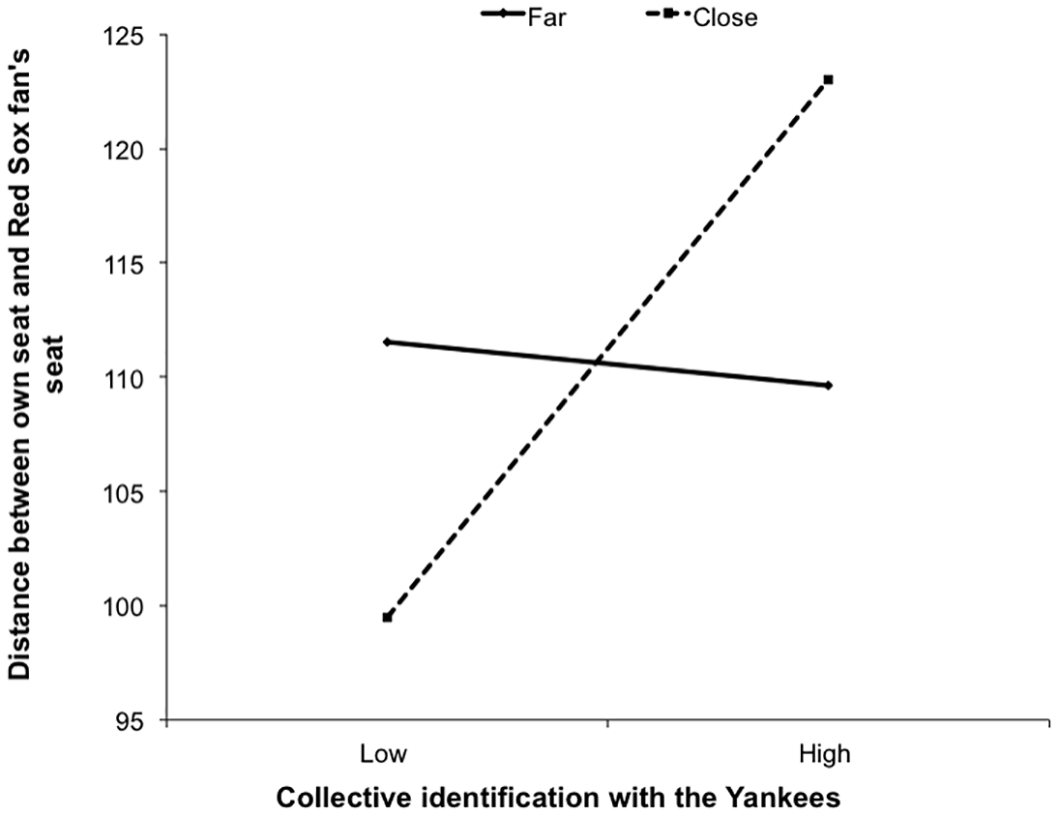
Distance between participants’ own chosen seat and their chosen seat for a Red Sox fan’s seat.

To examine whether our distance manipulation had an effect on participants’ desired seating distance from a Red Sox fan as a function of their collective identification with the Yankees, we conducted a multiple regression analysis. We calculated a composite collective identification score and an intergroup seating distance index for each participant. We regressed the intergroup seating distance on mean-centered collective identification, distance condition (far = 0; close = 1), and the interaction term, *F*(3, 232) = 2.96, *p* = .03, *R*^*2*^ = .04. As expected, the interaction between collective identification and the distance condition significantly predicted intergroup seating distance, *t*(232) = 2.10, *p* = .04, *B* = 5.63, 95% CI [.33, 10.93] (see Fig 6). Simple slopes analysis indicated that collective identification predicted seating proximity in the close condition, *t*(232) = 2.39, *p* = .08, *B* = 5.63, 95% CI [-.26, 5.04]; but not in the far condition, *t*(232) = -1.02, *p* = .31, *B* = -3.24, 95% CI [-9.53, 3.04]. Thus, Yankees fans who identified more strongly with their team wanted to sit farther away from Red Sox fans.

### Discussion

When baseball fans were faced with intergroup threat, group members who identified more strongly with their favorite team endorsed policies that favored the in-group over a threatening out-group [4]. To our knowledge, this provides the first evidence that physical distance between the in-group and a threatening out-group can elicit discrimination among highly identified group members. Specifically, the relationship between collective in-group identification and intergroup discrimination was stronger when the out-group appeared to be close versus far. When the out-group appeared to be far away, group members’ collective identification did not predict their desired seating distance from members of the threatening out-group. However, when the out-group was manipulated to appear physically close, stronger in-group identification predicted the desire to sit farther away from members of the threatening out-group. These results provided additional evidence to support the role of perceived distance as one potential process that can mediate the relationship between intergroup threat and discrimination.

## General Discussion

Intergroup threat is prevalent among many social groups, including racial, ethnic, and religious groups, immigrants and host countries, nations, organizations, and teams. From the Great Wall of China to Wall Street, there is extensive evidence that social groups are willing to expend considerable energy and money to remove threatening out-groups from physical proximity. A contemporary example is the Israeli Government’s controversial construction of Israeli West Bank Barrier—a barrier that Israeli Prime Minister Rabin stated was to “reach a separation between us and them” for the expressed purpose of Israeli security [67].

Although previous research has been devoted to establishing the antecedents and consequences of intergroup threat, it has largely overlooked how the judgment of physical properties shapes the relationship between intergroup threat and discrimination. In this paper, we examined the phenomenon of proximity under intergroup threat. To this end, we presented three studies to better understand whether (a) intergroup threat alters how people represent the world around them and (b) how physical proximity can increase intergroup conflict. In Studies 1 and 2, we examined ways to reduce the bias in physical distance judgment elicited by intergroup threat, and found that a secure (vs. permeable) physical intergroup barrier (Study 1) and an apology from a transgressing out-group (Study 2) reduced the threat-induced proximity. In a third study, an experimental induction of proximity to a threatening out-group strengthened the relationship between collective in-group identification and discriminatory attitudes and behavioral intentions. These studies extended previous research showing that groups imbued with threat appeared to be physical closer among highly identified individuals by showing that distance might mediate the relationship between intergroup threat and discrimination.

### Intergroup Barrier Attenuates Physical Proximity

In Study 1, we showed that group members’ perception of a strong versus weak intergroup barrier moderated the relationship between perceived threat and distance judgment. Americans’ perception of threat from Mexican immigration predicted estimated proximity to Mexico City only when the US-Mexico border was perceived to be weak. Thus, a strong intergroup barrier may not only serve as a physical barrier between social groups, but also a psychological protective function for group members faced with potential threat from a bordering group. A strong intergroup barrier eliminated the effect of intergroup threat on judgment of physical closeness.

Importantly, we are not claiming that putting up strong physical barriers between bordering groups is the best solution to intergroup threat. For instance, building walls can have short-term effects for reducing physical proximity induced by intergroup threat, but may introduce long-term problems in other aspects of intergroup relations. Therefore, we do not advocate strengthening border security between groups in all circumstances, since it may increase intergroup animosity.

### Intergroup Apology Attenuates Physical Proximity

We provided evidence that a strong physical barrier between the in-group and the threatening out-group protects against the physical closeness effects from intergroup threat and showed that psychosocial factors such as apology and positive pre-threat relations with the out-group can serve similar functions in eliminating the estimated physical closeness of the transgressing out-group. Instead of having to rely on constructing physical barriers between groups, there are more socially constructive ways to ameliorate the threat-induced proximity effects. Interestingly, forgiveness is usually conceptualized as a reduced motivation to seek revenge and to avoid the transgressor [34]. In other words, forgiveness should make the transgressing individual feel psychologically *closer*. However, positive feelings toward the out-group can make an out-group seem physically *farther*. These seemingly divergent predictions were resolved in our data.

We would like to note that we find that the quality of cross-group friendship matters when considering the effect of intergroup apology on distance perception. However, because our study did not include a control condition, it is difficult to know whether high or low contact (or both) with intergroup friends is responsible for the difference. Future research should aim to disentangle the specific process. It is also important to note that an out-group apology is by no means the panacea for intergroup threat. Intergroup threat is not a one-dimensional construct, and it encompasses a wide range of characteristics. For instance, in situations where intergroup threat is induced by a negative comparison with an out-group (e.g., status threat, as in current Study 1), apology should not be the recommended solution to attenuate the proximity effects as well as the behavioral consequences. Instead, the idiosyncrasy of the intergroup dynamics and the nature of the threat should always be considered when attempting to find a suitable solution.

### Distance Judgments Affect Intergroup Consequences

We also assessed the causal relationship between physical proximity and intergroup discrimination. We directly manipulated the proposed mediator—distance judgment—and assessed the consequences on policy measures designed to assess discrimination. When appearing to be physically close to the in-group, a threatening out-group elicited more expression of discriminatory attitudes toward members of this threatening group and made people choose to sit farther away from them. These findings pointed to the value of understanding the role distance judgment plays in intergroup relations.

Together, these studies help clarify the role of physical proximity in intergroup relations. Rather than perceiving the world as is, our motives, experiences, and expectations can modify how we experience external stimuli [10]. It has been proposed that these biased representations of the world may be more than just perceptual errors, but rather evolved adaptive biases that are beneficial to survival [68]. On the group level, biased information processing to promote vigilance and awareness of a threat from out-groups serves as a process that is advantageous to the survival and well being of one’s in-group. We found that these biases elicited discriminatory intergroup attitudes and behavioral intentions, offering an important process through which we could understand and predict intergroup outcomes. We suspect that these processes should extend beyond the realm of intergroup relations. To the extent that representation of physical stimuli can lead to action that benefit the survival and well-being of oneself, one’s affiliates, and one’s social group, it is fruitful to understand such biases in representation and judgment.

Although we focused on physical distance estimates, distance is just one lens through which this framework could operate [26]. For instance, we found that intergroup threat also exaggerated judgments of population size of the threatening out-group, especially among highly identified group members. This was analogous to our physical distance effects in that both indicated a looming effect of the threatening out-group—namely exaggerated physical proximity *and* size. There is reason to believe that intergroup concerns also shape other perceptual dimensions, ranging from mental state inferences [69] to skin tone judgments [70]. We also suspect that the influence of social identity extends beyond the visual system to other perceptual modalities [26].

We do not intend to claim judgment of physical stimuli, or in this case distance or size judgment, as the only process through which intergroup threat leads to discrimination. This research serves as a first step in conceptualizing intergroup threat as grounded in perceptual representation, and thus offers an alternative pathway for reducing the negative consequences of intergroup threat. Prior work in our field on intergroup threat has traditionally focused on its antecedents and consequences, whereas we sought to explain a perceptual process that underlies the relationship between intergroup threat and its consequences. Here we provided empirical evidence for the proposed relationships, as well as how factors related to the intergroup dynamic should flexibly change these relationships. It is, of course, unclear where in the processing stream these biases took hold. At present, we cannot conclude that is was grounded in early perceptual processing. Nevertheless, this approach will hopefully enable researchers to generate novel predictions in studying intergroup processes that are unique from predictions generated from merely focusing on the relationship between intergroup threat and its intergroup outcomes.

### Conclusion

Intergroup threat is prevalent among various social groups, and typically results in responses that serve protective functions for one’s social identity, including in-group favoritism, out-group derogation, self-stereotyping, and/or other forms of collective resistance [27]. In situations where it is difficult to reduce intergroup threat, understanding the psychological processes underlying intergroup threat and discrimination may enable us to alleviate discrimination, such as racism and ethnocentrism, via alternative routes—namely by acting on perception. For this reason, we call for more work testing and evaluating the role of physical proximity in intergroup relations, as well as work examining other potential processes underlying intergroup threat. This research may ultimately shed light on both historical and current real world phenomena, such as the construction of the Israeli West Bank Barrier, the Arizona immigration law, and many more.
